# Four Decades of Educational Inequalities in Hospitalization and Mortality among Older Swedes

**DOI:** 10.1371/journal.pone.0152369

**Published:** 2016-03-31

**Authors:** Jenny Torssander, Anders Ahlbom, Karin Modig

**Affiliations:** 1 The Swedish Institute for Social Research, Stockholm University, Stockholm, Sweden; 2 The Institute of Environmental Medicine, Karolinska Institutet, Stockholm, Sweden; University of Brescia, ITALY

## Abstract

**Background:**

The inverse association between education and mortality has grown stronger the last decades in many countries. During the same period, gains in life expectancy have been concentrated to older ages; still, old-age mortality is seldom the focus of attention when analyzing trends in the education-mortality gradient. It is further unknown if increased educational inequalities in mortality are preceded by increased inequalities in morbidity of which hospitalization may be a proxy.

**Methods:**

Using administrative population registers from 1971 and onwards, education-specific annual changes in the risk of death and hospital admission were estimated with complimentary log-log models. These risk changes were supplemented by estimations of the ages at which 25, 50, and 75% of the population had been hospitalized or died (after age 60).

**Results:**

The mortality decline among older people increasingly benefitted the well-educated over the less well-educated. This inequality increase was larger for the younger old, and among men. Educational inequalities in the age of a first hospital admission generally followed the development of growing gaps, but at a slower pace than mortality and inequalities did not increase among the oldest individuals.

**Conclusions:**

Education continues to be a significant predictor of health and longevity into old age. That the increase in educational inequalities is greater for mortality than for hospital admissions (our proxy of overall morbidity) may reflect that well-educated individuals gradually have obtained more possibilities or resources to survive a disease than less well-educated individuals have the last four decades.

## Introduction

Life expectancy is constantly increasing in the Swedish population as well as in other high- and middle income countries[1]. Sweden has been described as one of the “health leaders” with high life expectancy (ibid.), and compressed variation in the ages at which death occurs[2]. One key question–of concern for both public health policies and individuals’ quality of life–is whether these extra years of life expectancy are characterized by disease or not. For Sweden, the figure is fairly optimistic: Hospitalization, significant diseases, self-reported ill health, and activity limitations have been postponed to higher ages[3, 4]. On the other hand, years lived with acute and chronic diseases have declined more slowly than have mortality rates[5] and complex health problems increased among the oldest old in Sweden during the 1990s[6].

A second, less explored, key question is to what extent the life expectancy rise and the postponement of ill health and hospital admissions to higher ages have been equally distributed across socioeconomic strata. Educational disparities in life expectancy have increased the last decades, in Sweden [7] as well as in other Nordic countries [8]. Thirty years ago, 30-year-old Swedish women with a college degree could expect to live approximately two years longer than their less educated counterparts. In 2007, this disparity had grown to four years. For men, the increase was smaller but yet noticeable[7]. Increasing relative educational inequalities in mortality have been identified in several other high- and middle-income countries including Sweden[9–11]. However, as far as we know, it is less clear how educational disparities in the risk of dying have changed among older Swedes, even if most of the mortality reduction lately has occurred in this age group[12].

In comparison to the strengthening of the education-mortality association in middle age, social inequalities in self-reported health have been fairly stable in Sweden and several other countries the last decades[13]. Unchanging social gradients have been reported for various diseases and health-related symptoms[14], also among older people[15]. This may suggest that health and mortality disparities do not necessarily follow the same trends. Socioeconomic differences in health expectancy–or disability-free life expectancy–have however been suggested to be greater than life expectancy inequalities at age 65 years in several European countries [16].

Taken together, there are two gaps in the previous literature that need to be closed. First, although recent gains in life expectancy have been concentrated to older ages, trends in social inequalities in mortality risk and life expectancy have mostly been assessed for the working-aged or the younger old, even if a few exceptions exist[17]. We therefore shift the attention to later-life mortality where the mortality decline has been most substantial lately. Second, we do not know if increased social inequalities in mortality has come with increased inequalities in hospitalization–here used as a proxy for morbidity since most serious diseases are treated within hospitals and health care is universal in Sweden. If the advantaged groups, such as individuals with higher levels of education, have primarily gained life years with (or without) hospitalization may be important for the development of public health and social policies, and may also potentially affect our understanding of the increasing socioeconomic gaps in death risk. To show the trends for three educational groups in mortality and hospitalization from age 60 and onwards–and discuss them in conjunction–are therefore the objectives with the present study.

## Material and Methods

### Data, study population, and measures

We draw on population register information for individuals aged 60 years and older, born 1911–1940 and living in Stockholm, Uppsala, or Gävleborg County (covering around 20% of the Swedish population). The restriction to these three counties was made because hospital admission data for entire Sweden was only available from year 1987, while the selected counties had full coverage from year 1971 which made it possible to cover a longer time period (1971–2014). Those who moved to any of these counties after 60 years of age were excluded from the study population, and those who left any of the three counties for another part of Sweden before 1987, or for another country at any time during follow-up, were censored at the date of migration (4.2%).

Hospitalization was defined as the first hospital admission of at least one night after age 60 for any disease or event resulting in hospital admission. Information on date of death was collected from The Swedish Cause of Death Register (years 1971–2014), including deaths occurring in Sweden, or elsewhere if the individual was registered in Sweden.

Highest attainted educational level originated from the Census of 1970 or, if this information was missing, from the annual educational information in the Longitudinal Integration Database for Health Insurance and Labour Market Studies (for 4% of the study population) compiled by Statistics Sweden from 1990 and onwards. The following educational levels were distinguished (Table 1): 1) Compulsory/basic education; 2) Upper secondary education (vocational and academic tracks); and 3) Tertiary education (any college or university education). Individuals with no information on education were excluded from the study (N = 24351, 2.5%). The registers were linked together using individual-unique identification numbers after approval from the Regional Ethical Review Board in Stockholm (Dnr 2011/136-31/5) and the data were de-identified.

**Table 1:** Number (%) of the study population with basic, upper secondary and tertiary education at age 60 (at the time of study inclusion) by gender and time period.

Educational level	Period			
	*1971–1980*	*1981–1990*	*1991–2000*	*Total*
*Men*				
Basic education	70 919 (59.2%)	50 586 (50.5%)	39 925 (43.0%)	161 430 (51.6%)
Upper secondary education	36 254 (30.3%)	35 435 (35.4%)	36 008 (38.8%)	107 697 (34.4%)
Tertiary education	12 539 (10.5%)	14 204 (14.2%)	16 855 (18.2%)	43 598 (14.0%)
*Total*	119 712 (100%)	100 225 (100%)	92 788 (100%)	312 725
*Women*				
Basic education	92 789 (70.7%)	70 626 (64.5%)	50 105 (53%)	213 520 (63.6%)
Upper secondary education	31 158 (23.7%)	29 114 (26.5%)	30 031 (31.7%)	90 303 (26.9%)
Tertiary education	7 372 (5.6%)	9 807 (9%)	14 495 (15.3%)	31 674 (9.5%)
*Total*	131 319 (100%)	109 547 (100%)	94 631 (100%)	335 497

### Statistical analyses

The annual age-specific risks of first hospital admission and death were calculated for all ages above 60 and for the years 1971 to 2014. The risks were computed as the number of events per year divided with the number of individuals at risk in the beginning of the year. The distribution of the age-specific risks for a calendar year was calculated by multiplying the risks with the probability of being alive/free of hospitalization at each age. We then derived the ages at which 25, 50 (i.e., the median age of death/first hospital admission), and 75% of the study population had been subject to hospital admission or death. All calculations were under the condition of survival up until 60 years of age.

To further examine education-specific trends in hospitalization and death risks, we fitted a discrete time logistic model with a complementary log-log link[18] including an interaction term between birth year and education. The relative risk of being born one year later (the annual change over time) was estimated at each year, based on age at death or first hospital admission–treated as a categorical variable–and used as a time-varying predictor of the outcome in the regression model. The age-adjusted relative risk (RR) with 95% confidence intervals was measured for men and women separately and stratified by age and period. The percentage (%) annual change in the risk was estimated by subtracting one from the relative change (RR −1). The annual change represents a relative change to the starting value for each education strata, without providing the size of the absolute differences. Therefore, we also calculated risk differences (subtracting the absolute risks) and relative risks (the risk for the lowest educated divided with the risk for the highest educated). We further derived remaining life expectancy at the age of 60. Since the data structure does not include all ages in all time periods that we studied, life expectancy was averaged for years 2000 to 2014, i.e., a period where all ages were represented.

Because of the secular rise in education over time (Table 1), selection as well as the meaning of different educational levels have changed and any compositional changes may affect the results. As a robustness test, we controlled for the share with basic education in each respondents birth cohort, i.e., holding the proportion in the population with a certain education level constant [19]. As a further sensitivity test, we repeated all analyses for Swedish-born individuals only to ensure that the results were not affected by unreliable information about educational level and/or hospital admission/mortality for the foreign-born part of the study population (any deviations reported).

## Results

### Mortality

**Fig 1:**
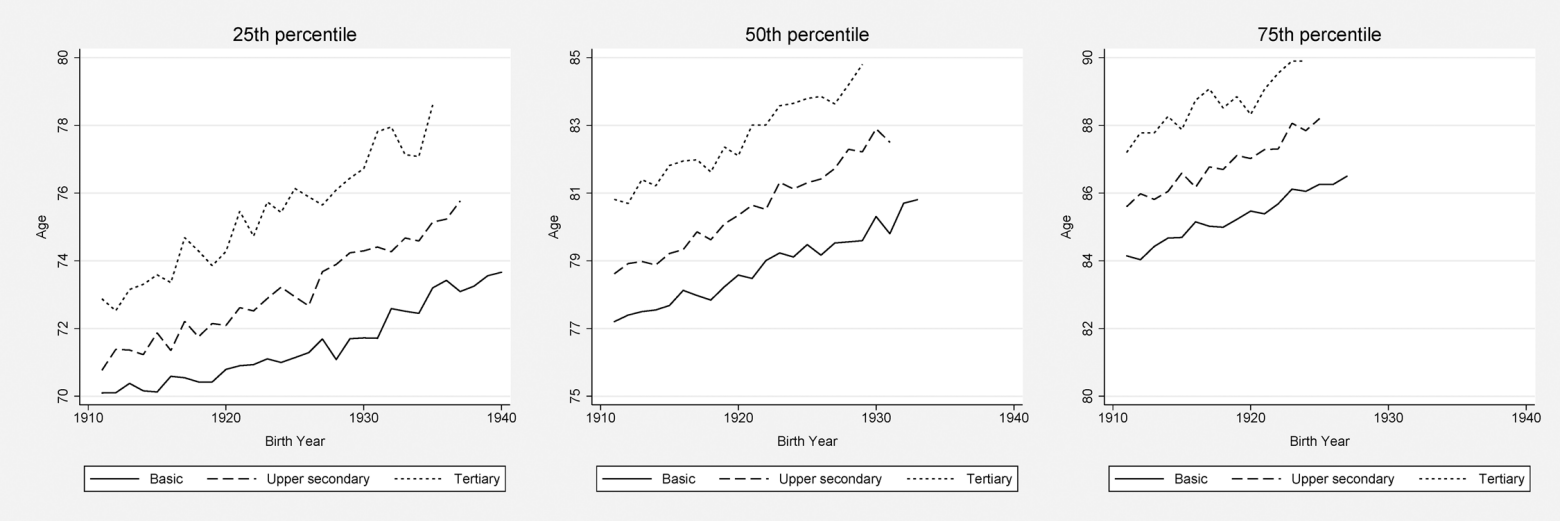
Survival by education, men. Age at which 25, 50 (i.e., median age of death), and 75% of the study population had died.

**Fig 2:**
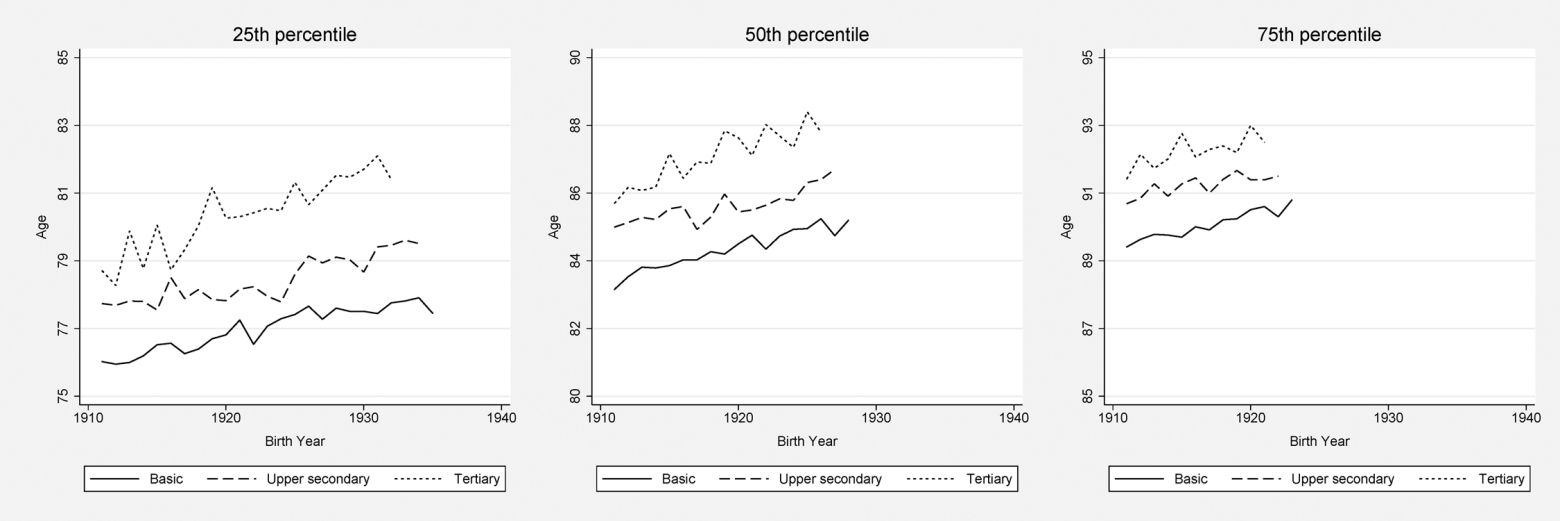
Survival by education, women. Age at which 25, 50 (i.e., median age of death), and 75% of the study population had died.

The ages at which 25, 50, and 75% of the population had died in each birth cohort were, as expected, postponed to older ages over time (Figs 1 and 2). Such a development took place in all educational groups for both men and women. The shift to greater inequalities was clearer in the left end of the mortality distribution, i.e., in the 25^th^ compared to the 50^th^ and the 75^th^ percentile. For the 25^th^ percentile, the absolute difference between men with basic education and tertiary education rose from 2.8 years for the 1911^th^ birth cohort to 5.4 years for the 1935^th^ birth cohort. The corresponding figures for women were 2.7 years (for birth year 1911) and 3.6 years (birth year 1932).

For lower educated men, the age when 25% had died was 70.1 years for those born 1911 and 73.2 years for those born in 1935. For higher educated men, 25% had died at age 72.9 years (birth cohort 1911) and 78.6 years (birth cohort 1935). Thus, among the younger old men, the well-educated clearly experienced a greater mortality decline than the less well-educated did. For women, the inequality rise was less remarkable in the 25^th^ percentile; from 76 to 77.8 years for those with basic education, and from 78.7 to 81.4 years for women with tertiary education (here comparing the birth cohort of 1911 to the birth cohort of 1932).

The education-specific trend for the median age of death (i.e., the 50^th^ percentile) was fairly parallel for the highest and the lowest educational categories among women. For men, however, the educational gap for the 50^th^ percentile increased with 1.6 years between basic and tertiary education (from birth year 1911 to birth year 1929). For the 75^th^ percentile, we found slightly growing disparities for men (of 0.7 years), but, again, more parallel trends for different education strata among women, indicating stable absolute inequalities.

Tables 2 and 3 show the average change (%) per calendar year in one-year death risks by age group and period. The regression estimates indicate that there has been a significant unequal development in death risk over time by education. On average, the annual decrease in death risk after age 60 was 2% for women with tertiary education compared to 1% for women with basic education (Table 3, last column). The corresponding estimates for men were 2.8% and 1.6%, respectively (Table 2, last column). The size of the annual change for individuals with upper secondary education was between the size of the change for basic and tertiary education.

**Table 2:** Average annual change in one year death risk, in per cent (95% CI) from clog-log models by age group and period, men.

Age group	Period				
	*I*: *1971–1980*	*II*: *1981–1990*	*III*: *1991–2000*	*IV*: *2001–2014*	*All periods*
*I*: *60–69 years*					
Basic education	1.4 (0.4, 2.4)	-1.1 (-1.5, -0.5)	-1.8 (-2.4, -1.3)	-1.8 (-3.0, -0.7)	
Upper secondary education	-1.0 (-2.0, 0.1)	-2.3 (-2.8, -1.8)	-2.8 (-3.4, -2.3)	-2.7 (-3.9, -1.6)	
Tertiary education	-4.8 (-6.1, -3.4)	-4.5 (-5.1, -4.0)	-4.2 (-4.8, -3.7)	-3.8 (-4.9, -2.6)	
*II*: *70–79 years*					
Basic education		-1.0 (-1.7, -0.3)	-1.7 (-2.0, -1.3)	-1.8 (-2.1, -1.5)	
Upper secondary education		-2.1 (-2.8, -1.4)	-2.4 (-2.8, -2.1)	-2.4 (-2.7, -2.2)	
Tertiary education		-3.1 (-3.8, -2.3)	-3.3 (-3.7, -2.9)	-3.1 (-3.4, -2.9)	
*III*: *80–89 years*					
Basic education			-1.2 (-1.8, -0.5)	-1.5 (-1.7, -1.3)	
Upper secondary education			-1.7 (-2.3, -1.1)	-1.9 (-2.2, -1.7)	
Tertiary education			-2.4 (-3.0, -1.8)	-2.5 (-2.7, -2.2)	
*IV*: *90–99 years*					
Basic education				-0.7 (-1.2, -0.3)	
Upper secondary education				-1.0 (-1.4, -0.5)	
Tertiary education				-1.2 (-1.7, -0.8)	
*All ages*					
Basic education					-1.6 (-1.6, -1.5)
Secondary education					-2.2 (-2.2, -2.1)
Tertiary education					-2.8 (-2.9, -2.8)

**Table 3:** Average annual change in one year death risk, in per cent (95% CI) from clog-log models by age group and period, women.

Age group	Period				
	*I*: *1971–1980*	*II*: *1981–1990*	*III*: *1991–2000*	*IV*: *2001–2014*	*All periods*
*I*: *60–69 years*					
Basic education	1.7 (0.3, 3.0)	-1.0 (-1.6, -0.3)	-1.2 (-1.9, -0.5)	-1.4 (-2.9, 0.1)	
Upper secondary education	-0.6 (-2.0, 0.9)	-1.9 (-2.5, -1.2)	-1.8 (-2.6, -1.1)	-2.0 (-3.5, -0.5)	
Tertiary education	-3.6 (-5.8, -1.3)	-3.6 (-4.4, -2.8)	-3.0 (-3.7, -2.2)	-3.1 (-4.6, -1.6)	
*II*: *70–79 years*					
Basic education		-0.4 (-1.2, 0.5)	-1.3 (-1.8, -0.9)	-1.3 (-1.7, -1.0)	
Upper secondary education		-1.7 (-2.5, -0.8)	-1.9 (-2.4, -1.5)	-1.8 (-2.2, -1.5)	
Tertiary education		-2.4 (-3.4, -1.4)	-3.1 (-3.6, -2.6)	-2.7 (-3.0, -2.3)	
*III*: *80–89 years*					
Basic education			-1.1 (-1.8, -0.5)	-1.1 (-1.3, -0.9)	
Upper secondary education			-1.7 (-2.3, -1.0)	-1.5 (-1.7, -1.3)	
Tertiary education			-2.2 (-2.8, -1.6)	-1.9 (-2.1, -1.7)	
*IV*: *90–99 years*					
Basic education				-0.5 (-0.9, -0.2)	
Upper secondary education				-0.7 (-1.1, -0.4)	
Tertiary education				-0.9 (-1.3, -0.6)	
*All ages*					
Basic education					-1.0 (-1.1, -0.9)
Upper secondary education					-1.4 (-1.5, -1.4)
Tertiary education					-2.0 (-2.1, -1.9)

The pattern of increased educational inequalities in death risk (measured as the annual change) was found in most ages, although particularly so in the lower age span of our study population. In the youngest age group, 60–69 years of age, we could even observe a significant rise in the annual change in death risk for men and women with basic education in the 1970ies (on average 1.4% for men and 1.7% for women). The educational gap in the death risk change also grew less in more recent time periods compared to three or four decades ago. Yet, confidence intervals were sometimes overlapping in the age- and period-specific results, and particularly so between adjacent educational categories (basic vs. secondary and secondary vs. tertiary). Besides, we could only make a perfect comparison by both age and period in our latest study period (2001–2014) or for the youngest age group (60–69 years). In sum, however, our results for mortality suggest that the differences between educational groups are the largest–and the inequality increase most intense–in relatively early mortality.

Although our regression estimates illustrate educational inequalities in terms of the rate of improvement, they are less informative when it comes to the magnitude of absolute inequalities. To compensate for this, we derived absolute risks for some age groups and years. The risk difference between a 69-year-old man with basic education compared to his college-educated counterpart was 1.4% in 2010. For women, the corresponding difference was 0.1%. At age 79, the corresponding difference was 2.4% for men and 1.1% for women; and at age 89, 4.5% for men and 1.9% for women.

Yet another way to show the magnitude of inequalities is to derive differences in remaining life expectancy. In the latter part of our study period (2000–2014), the remaining life expectancy at age 60 was 20.7 years for men with basic education and 24.5 years for men with tertiary education. The corresponding figures for women were 24.7 and 27.7 years, respectively.

### Hospital admissions

**Fig 3:**
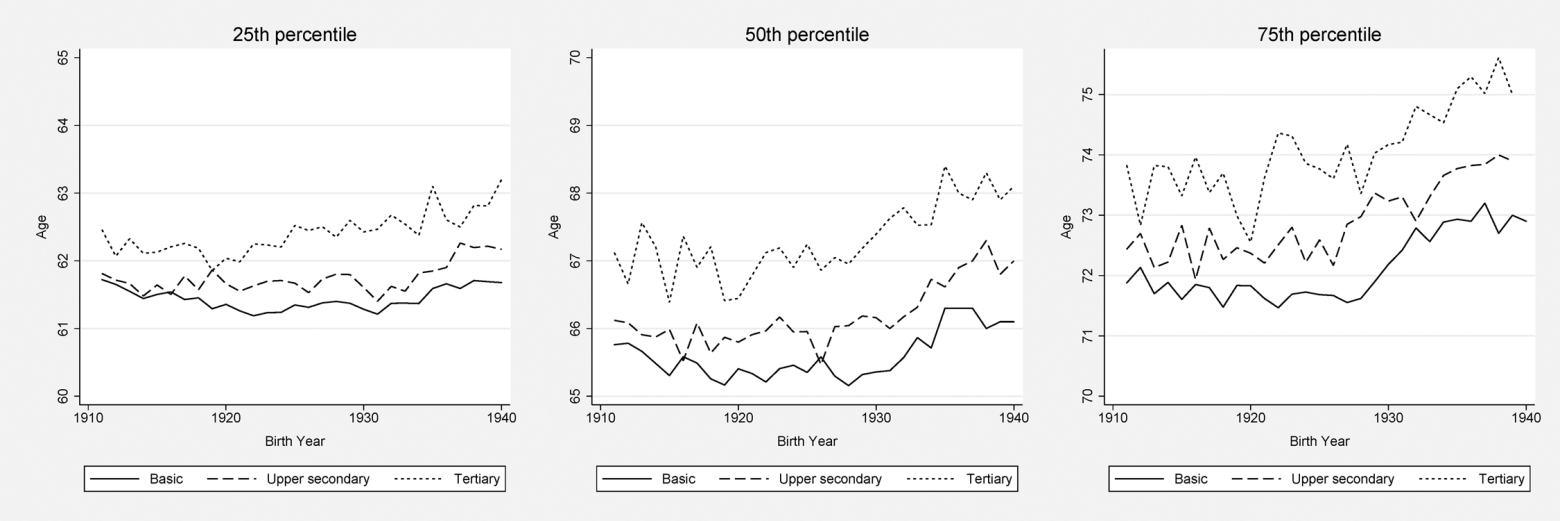
Hospitalization by education, men. Age at which 25, 50 (i.e., median age of death), and 75% of the study population had been admitted to hospital.

**Fig 4:**
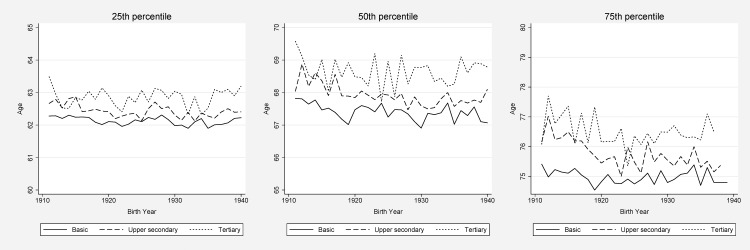
Hospitalization by education, women. Age at which 25, 50 (i.e., median age of death), and 75% of the study population had been admitted to hospital.

Figs 3 and 4 show the education-specific ages at which 25, 50, and 75% of the study population had been admitted to hospital for the first time after age 60. Each year, and for all three percentiles, the well-educated were older when they were first admitted to hospital after 60 years of age compared to those with basic education, but there was quite some degree of yearly volatility. The age differences in the first hospital admission between women with tertiary and basic education were fairly constant when comparing birth cohorts 1911 and 1940 for the 25^th^ and 50^th^ percentile, corresponding to a 1-year age difference between the highest and the lowest educational levels for the 25^th^ percentile, and about 2 years difference for 50^th^ percentile. Among men, however, an increase of slightly more than half a year in the educational gap could be discerned in these percentiles.

At the age when 75% had been admitted to hospital, the gap between the highest and lowest educational groups widened with about 1 year for women if we compare the birth cohorts of 1911 and 1940. However, examining the overall pattern across the full period (Fig 4), a tendency of increased inequalities was not evident between the lower and higher educated strata, although a widening gap between individuals with upper secondary education and college education could be seen. For men, the educational differences stayed rather constant at around 2 years in the 75^th^ percentile. Generally, the absolute age differences were smaller for the 25^th^ percentile than for the 50^th^ and the 75^th^ percentile (compared to mortality were the largest differences often were found in the left end of the mortality distribution).

Tables 4 and 5 show the annual changes in the yearly risk of a first hospital admission after age 60 (in %). The changes in the risk of hospital admission over time were smaller than the death risk changes; yet, the educational differences in the age of a first hospital admission did also grow over time according to the regression estimates. Individuals with tertiary education experienced a greater annual decline (*alt*. a smaller increase) in hospitalization risk than those with secondary or basic educational levels. The overall annual decreases amounted to 0.3% for men with basic education, 0.5% decrease for men with upper secondary education, and a 0.9% decrease for men with tertiary education. Consistent with the descriptive statistics, the educational gap widened primarily among the younger old: Non-overlapping confidence intervals between basic and tertiary education were primarily found in the age group 60–69 years, and the difference in the annual risk change weakened over time in this age group (Table 4). For the oldest old men, the risk of hospitalization increased but the increase was not statistically significant and there were no difference between education strata.

**Table 4:** Average annual change in hospital admissions, in per cent (95% CI), estimated from clog-log models by age group and period, men.

Age group	Period				
	*I*: *1971–1980*	*II*: *1981–1990*	*III*: *1991–2000*	*IV*: *2001–2014*	*All periods*
*I*: *60–69 years*					
Basic education	1.5 (1.1, 2.0)	-0.3 (-0.6, -0.1)	-1.8 (-2.1, -1.5)	0.8 (0.0, 1.5)	
Upper secondary education	0.5 (0.1, 1.0)	-0.9 (-1.1, -0.6)	-2.3 (-2.5, -2.0)	0.5 (-0.2, 1.3)	
Tertiary education	-1.6 (-2.2, -0.9)	-1.8 (-2.1, -1.5)	-2.8 (-3.0, -2.5)	0.2 (-0.5, 0.9)	
*II*: *70–79 years*					
Basic education		-1.2 (-1.9, -0.5)	-1.7 (-2.1, -1.2)	-0.2 (-0.5, 0.1)	
Upper secondary education		-1.5 (-2.2, -0.8)	-1.8 (-2.2, -1.4)	-0.4 (-0.7, -0.1)	
Tertiary education		-2.0 (-2.7, -1.2)	-2.1 (-2.5, -1.7)	-0.5 (-0.8, -0.2)	
*III*: *80–89 years*					
Basic education			-1.8 (-3.0, -0.6)	-0.4 (-0.9, 0.1)	
Upper secondary education			-2.0 (-3.2, -0.8)	-0.5 (-1.0, -0.1)	
Tertiary education			-2.4 (-3.6, -1.1)	-0.7 (-1.1, -0.2)	
*IV*: *90–99 years*					
Basic education				0.8 (-1.0, 2.6)	
Upper secondary education				0.8 (-1.0, 2.6)	
Tertiary education				0.9 (-0.9, 2.8)	
*All ages*					
Basic education					-0.3 (-0.4, -0.3)
Upper secondary education					-0.6 (-0.7, -0.6)
Tertiary education					-0.9 (-1.0, -0.9)

**Table 5:** Average annual change in hospital admissions, in per cent (95% CI), estimated from clog-log models by age group and period, women.

Age group	Period				
	*I*: *1971–1980*	*II*: *1981–1990*	*III*: *1991–2000*	*IV*: *2001–2014*	*All periods*
*I*: *60–69 years*					
Basic education	0.9 (0.4, 1.3)	-0.8 (-1.0, -0.5)	-0.6 (-0.9, -0.3)	1.2 (0.4, 1.9)	
Upper secondary education	-0.3 (-0.8, 0.2)	-1.2 (-1.5, -0.9)	-0.8 (-1.1, -0.5)	1.0 (0.3, 1.8)	
Tertiary education	-1.6 (-2.4, -0.8)	-1.7 (-2.0, -1.3)	-1.2 (-1.5, -0.9)	0.8 (0.0, 1.5)	
*II*: *70–79 years*					
Basic education		-1.2 (-1.9, -0.6)	-0.5 (-0.9, -0.1)	0.5 (0.2, 0.8)	
Upper secondary education		-1.8 (-2.4, -1.1)	-0.7 (-1.1, -0.3)	0.4 (0.1, 0.6)	
Tertiary education		-2.2 (-2.9, -1.4)	-0.9 (-1.3, -0.5)	0.2 (0.0, 0.5)	
*III*: *80–89 years*					
Basic education			-0.8 (-1.8, 0.1)	-0.1 (-0.4, 0.3)	
Upper secondary education			-0.9 (-1.8, 0.0)	-0.1 (-0.5, 0.3)	
Tertiary education			-1.1 (-2.0, -0.1)	-0.3 (-0.6, 0.1)	
*IV*: *90–99 years*					
Basic education				0.4 (-0.7, 1.5)	
Upper secondary education				0.2 (-0.9, 1.3)	
Tertiary education				0.3 (-0.8, 1.5)	
*All ages*					
Basic education					0.2 (0.1, 0.2)
Upper secondary education					0.0 (-0.1, 0.0)
Tertiary education					-0.3 (-0.3, -0.2)

The most educated women overall experienced an annual decline of 0.3%, while women with basic education increased the risk of hospitalization of about 0.2% per year (last row, Table 5), i.e. corresponding to a difference of 0.5 percentage points in the average annual change. The hospitalization risk for women with secondary education did not change over time. Increased educational inequalities in hospitalization among women were primarily detected among the youngest old in the beginning of our study period. Among the oldest old (90–99 years), the estimates were of similar size for all educational groups.

To get an indication of the absolute inequality levels, it can be mentioned that the risk differences (tertiary-basic education) for a first hospital admission for our example persons (a 69-year-old man/woman in 2010) were 2.7% (men) and 0.5% (women). At age of 79 years, the corresponding differences were 2.2% (men) and 1.9% (women).

Finally, we adjusted for the proportion of people with basic education in the individual’s own birth cohort in order to capture if compositional changes could be driving the observed trends in hospitalization or mortality (not in Tables). The educational gradients were almost identical to the gradients in the unadjusted models, but the estimates were less precise.

## Discussion

Education has for a long time been recognized as a strong predictor of health and longevity, and recent studies suggest that educational inequalities in premature mortality are increasing in Sweden as well as in many other European countries[9], while health inequalities have been more stable[13]. We looked closer at the education–morbidity/mortality association during the last four decades in Sweden among people aged 60 years and older, for different age groups and parts of the distribution of mortality and the first hospital admission which may reflect the initial shift from health to morbidity. Our main findings are:

*First*, we found increased educational inequalities in death risks among older Swedes over time. This increase was most pronounced in the lower age span of our study population, i.e., for relatively early mortality. The annual mortality change continued to be of advantage to the well-educated over the less well-educated into older ages, particularly for men. For women, absolute inequalities seemed to be fairly constant in the right end of the mortality distribution. We further found that, among older individuals, all educational groups could expect postponed mortality from 1980 and onwards (during the 1970s, individuals with basic education actually experienced an increased mortality risk). Combining our results with known trends in the educational gap for middle-aged individuals, we conclude increasing educational inequalities exist both in younger and older ages, and that survival improvements tend to benefit the well-educated more than their less well-educated counterparts over time.

*Second*, educational inequalities seem to grow faster for mortality than for morbidity, although educational inequalities in hospitalization increased somewhat: A higher level of education was associated with a greater delay in (the first) hospital admission to older ages over time. However, the gap was constant for the oldest old, where no inequality increase took place. Our results further suggested that the educational differences in the postponement in the age when the first hospital admission occurred were not vast, usually less than a year, but yet not negligible. We are aware that a hospital admission is not necessarily equivalent to a health or morbidity measure. However, because most serious diseases are taken care of within hospitals (although the hospital stays may have become shorter over time)–and health care is universal and almost free of charge in Sweden–we believe that hospitalization is a decent proxy of morbidity. Still, interpretations that place hospitalization on par with ill-health should be made with caution, and if so, as a crude proxy of incidence rather than prevalence since we only studied the first hospital admission after age 60.

*Third*, during the 20^th^ century, average levels of education have risen in Sweden as well as elsewhere. Declining shares of low-educated people may imply that this group have become increasingly negatively selected on characteristics important to health and longevity (or changing health-selection into higher education). This is potentially problematic, but our finding suggests, however, that compositional changes did not explain increasing educational inequalities in survival or hospitalization. This is in agreement with previous studies equalizing for compositional shares[19–20]. The stability in educational differences in hospitalization also remained after this control. However, the educational opportunities beyond compulsory education were still rather limited for most birth cohorts included in the present study, and a strong negative selection is therefore perhaps unlikely. Consequences of distributional changes in education may be more influential for later birth cohorts where secondary and tertiary educational levels are more expected. Yet, the well-educated may become less positively selected which could mitigate a trend of increasing inequalities.

## Conclusions

To sum up, educational inequalities in morbidity and mortality do not fully seem to change in concert among older individuals. The increase in educational inequalities in hospitalization has been less intense, while the education-mortality gap has grown faster, particularly among men. After age 90, educational differences in hospitalization did not change at all. Socioeconomic influences may have some countervailing effects on hospitalization risks: Although an advantaged socioeconomic position is associated with less probability of disease, people in these positions may be more prone to seek health care for a given health condition, even if health care is universal and almost free of charge in Sweden. Diverging inequality trends for mortality and hospitalization may indicate that the health care needs of less educated people are not fully met, and that any discrepancy between educational groups in meeting needs has grown over time[21]. Another possibility is that highly educated individuals have obtained rising possibilities to survive after the incidence of a disease to a greater extent than individuals with fewer years of schooling[22]. Whether this depends on health care utilization or is primarily caused by other factors, such as larger social disparities in risk factors or increased economic and labor market inequalities in Sweden the last decades, is however a question for future research.
